# Identification of Potential Molecular Mechanism Related to Infertile Endometriosis

**DOI:** 10.3389/fvets.2022.845709

**Published:** 2022-03-28

**Authors:** Xiushen Li, Li Guo, Weiwen Zhang, Junli He, Lisha Ai, Chengwei Yu, Hao Wang, Weizheng Liang

**Affiliations:** ^1^Department of Obstetrics and Gynecology, Shenzhen University General Hospital, Shenzhen, China; ^2^Guangdong Key Laboratory for Biomedical Measurements and Ultrasound Imaging, School of Biomedical Engineering, Shenzhen University Health Science Center, Shenzhen, China; ^3^Shenzhen Key Laboratory, Shenzhen University General Hospital, Shenzhen, China; ^4^School of Pharmaceutical Sciences, Health Science Center, Shenzhen University, Shenzhen, China; ^5^Department of Pediatrics, Shenzhen University General Hospital, Shenzhen, China; ^6^Department of Teaching and Research, Shenzhen University General Hospital, Shenzhen, China; ^7^School of Future Technology, University of Chinese Academy of Sciences, Beijing, China; ^8^Chinese Academy of Sciences (CAS) Key Laboratory of Genome Sciences and Information, Beijing Institute of Genomics, Chinese Academy of Sciences, Beijing, China

**Keywords:** infertile endometriosis, molecular mechanism, GEO, bioinformatics, ceRNA

## Abstract

**Objectives:**

In this research, we aim to explore the bioinformatic mechanism of infertile endometriosis in order to identify new treatment targets and molecular mechanism.

**Methods:**

The Gene Expression Omnibus (GEO) database was used to download MRNA sequencing data from infertile endometriosis patients. The “limma” package in R software was used to find differentially expressed genes (DEGs). Weighted gene co-expression network analysis (WGCNA) was used to classify genes into modules, further obtained the correlation coefficient between the modules and infertility endometriosis. The intersection genes of the most disease-related modular genes and DEGs are called gene set 1. To clarify the molecular mechanisms and potential therapeutic targets for infertile endometriosis, we used Gene Ontology (GO), Kyoto Gene and Genome Encyclopedia (KEGG) enrichment, Protein-Protein Interaction (PPI) networks, and Gene Set Enrichment Analysis (GSEA) on these intersecting genes. We identified lncRNAs and miRNAs linked with infertility and created competing endogenous RNAs (ceRNA) regulation networks using the Human MicroRNA Disease Database (HMDD), mirTarBase database, and LncRNA Disease database.

**Results:**

Firstly, WGCNA enrichment analysis was used to examine the infertile endometriosis dataset GSE120103, and we discovered that the Meorangered1 module was the most significantly related with infertile endometriosis. The intersection genes were mostly enriched in the metabolism of different amino acids, the cGMP-PKG signaling pathway, and the cAMP signaling pathway according to KEGG enrichment analysis. The Meorangered1 module genes and DEGs were then subjected to bioinformatic analysis. The hub genes in the PPI network were performed KEGG enrichment analysis, and the results were consistent with the intersection gene analysis. Finally, we used the database to identify 13 miRNAs and two lncRNAs linked to infertility in order to create the ceRNA regulatory network linked to infertile endometriosis.

**Conclusion:**

In this study, we used a bioinformatics approach for the first time to identify amino acid metabolism as a possible major cause of infertility in patients with endometriosis and to provide potential targets for the diagnosis and treatment of these patients.

## Introduction

Endometriosis, an estrogen-dependent disease, is characterized by the appearance of the endometrium outside the uterus (1). Laparoscopy is the most common approach for diagnosing endometriosis (2). Treatment methods include surgery and oral hormonal drugs, but both of which are usually accompanied by side effects. Although the theories of implantation, somatic epithelial metaplasia, and induction help to explain the pathophysiology of endometriosis to some extent, the mechanism of endometriosis formation remains unknown. Endometriosis can induce symptoms such as dysmenorrhea, dysuria, fatigue, deep intercourse pain, and infertility, which can seriously affect the patient's body, work, life, and psychology (3–5). About 30–50% of patients with endometriosis will be accompanied by infertility (6). However, the mechanism of infertile endometriosis is still unclear. Despite the fact that infertile endometriosis has a significant impact on patients, our knowledge of the condition is still restricted.

As a system biology tool, Weighted Gene Co-expression Network Analysis (WGCNA) can be used to analyze the gene expression patterns of multiple samples and cluster genes with similar expression patterns. WGCNA can explore the relationship between diseases and gene modules, which act as significant markers and signaling pathways in the occurrence and development of diseases (7, 8). Therefore, WGCNA is frequently employed to investigate the molecular mechanisms of various diseases including coronary artery disease, neuropathic pain, colon adenocarcinoma, and acute myocardial infarction (9–12).

Therefore, in this research, we employed WGCNA to investigate the gene modules that caused infertile endometriosis and further explored the signaling pathways and competing endogenous RNA (ceRNA) regulatory networks involved in pathogenesis through bioinformatics. Our findings may provide new molecular mechanisms and therapeutic targets for the therapy of infertile endometriosis.

## Materials and Methods

### Data Filtered and Download

We used the keywords “endometriosis” and “infertility” to search the Gene Expression Omnibus (GEO) database (http://ncbi.nlm.nih.gov/geo/). The following conditions were used as screening criteria for this study: (1) The data set must include both fertile and infertile endometriosis patients, with a minimum of 5 patients in each group; (2) The type of tissue used for sequencing should be consistent; (3) The data set included in this study must contain the original sequencing data. Finally, the GSE120103 data set was screened out for further investigation.

### DEGs Identification and Bioinformatics Analysis

Only genes with adjusted *p* < 0.001 and |log2FC|> 2 were considered DEGs in this investigation, we searched the GSE120103 data set by the “limma” package in R software. The “clusterProfiler” package in R software was used for Gene Ontology. (GO) enrichment analysis and Kyoto Encyclopedia of Genes and Genomes (KEGG) enrichment analysis of DEGs. We displayed the results of the enrichment analysis through the “ggplot2” package in R software. We used the STRING database (https://cn.string-db.org/) to generate the protein-protein interaction (PPI) network where the gene interaction score was more than 0.9.

### WGCEA

WGCNA can integrate the results of sequencing into biologically significant co-expressed gene modules and analyze the correlation between these gene modules and diseases (12). Therefore, we used WGCNA to explore modules related to infertile endometriosis. We used the “WGCNA” package in R software to perform WGCNA analysis on the GSE120103 data set. we utilized the “pickSoftThreshold” function to filter the soft powers in this study. The topological overlap matrix (TOM) and the accompanying dissimilarity matrix (1-TOM) were then obtained based on the adjacency matrix's construction. The “cutreeDynamic” function in R software was used to identify different gene modules. Furthermore, the “moduleEigengenes” function and the “mergeCloseModules” function in R software were used to cluster and merge gene modules, respectively. Finally, the correlation coefficient between the modular characteristic gene and the disease was calculated.

### Identification the Shared Gene and Bioinformatics Analysis

We chose the MEorangered1 module because it had the strongest association with infertile endometriosis. DEGs and the genes in MEorangered1 were intersected. The Venn diagram was used to represent these intersecting genes, which were referred to as gene set 1. GO enrichment analysis and KEGG enrichment analysis were performed on gene set 1 by using R software. PPI networks were constructed though the STRING database. Cytoscape is a professional biological data analysis program often used to visualize PPI networks. In this study, we used the “CytoNCA” plug-in in Cytoscape software to calculate the Betweenness, Closeness, Degree, Eigenvector, Network, and Local Average Connectivity between nodes. Hub genes were defined as nodes with values above the median of these features. The PPI network between Hub genes was visualized using Cytoscape software.

### Identify of microRNA and lncRNA

MicroRNA(miRNA) has the ability to promote or inhibit mRNA degradation and translation. We searched for infertility-related miRNAs through the Human MicroRNA Disease Database (HMDD) (http://www.cuilab.cn/hmdd/) and the MiRTarBase database (https://mirtarbase.cuhk.edu.cn/m~iRTarBase/miRTarBase_2022/php/index.php) and the online tool LncBase Experimental v.2 (http://diana.imis.athena-innovation.gr/DianaTools/index.php?r=site/page&view=software) to find mRNAs and lncRNAs associated with these miRNAs. To search for lncRNAs associated with infertility, We used the LncRNADisease database (http://www.cuilab.cn/lncrnadisease) constructed by Peking University.

### Construction of ceRNA Regulate Network

We took intersections of target genes for each miRNA, DEGs and genes in the MEorangered1 module, respectively. The Degree value of each node in the ceRNA network was calculated using the cytoHubba plug-in in the cytoscape software. The ceRNA regulate network was visualized using Cytoscape software.

## Results

### Information of the GEO Dataset

We identified 18 datasets in the GEO database using the keywords “endometriosis” and “infertility.” According to our selection criteria, only the GSE120103 dataset, based on the GPL6480 sequencing platform, was retrieved. We used endometrial tissue sequencing data from 9 infertile and 9 fertile patients with endometriosis for bioinformatic analysis in this study.

### Identification of DEGs

**Figure 1:**
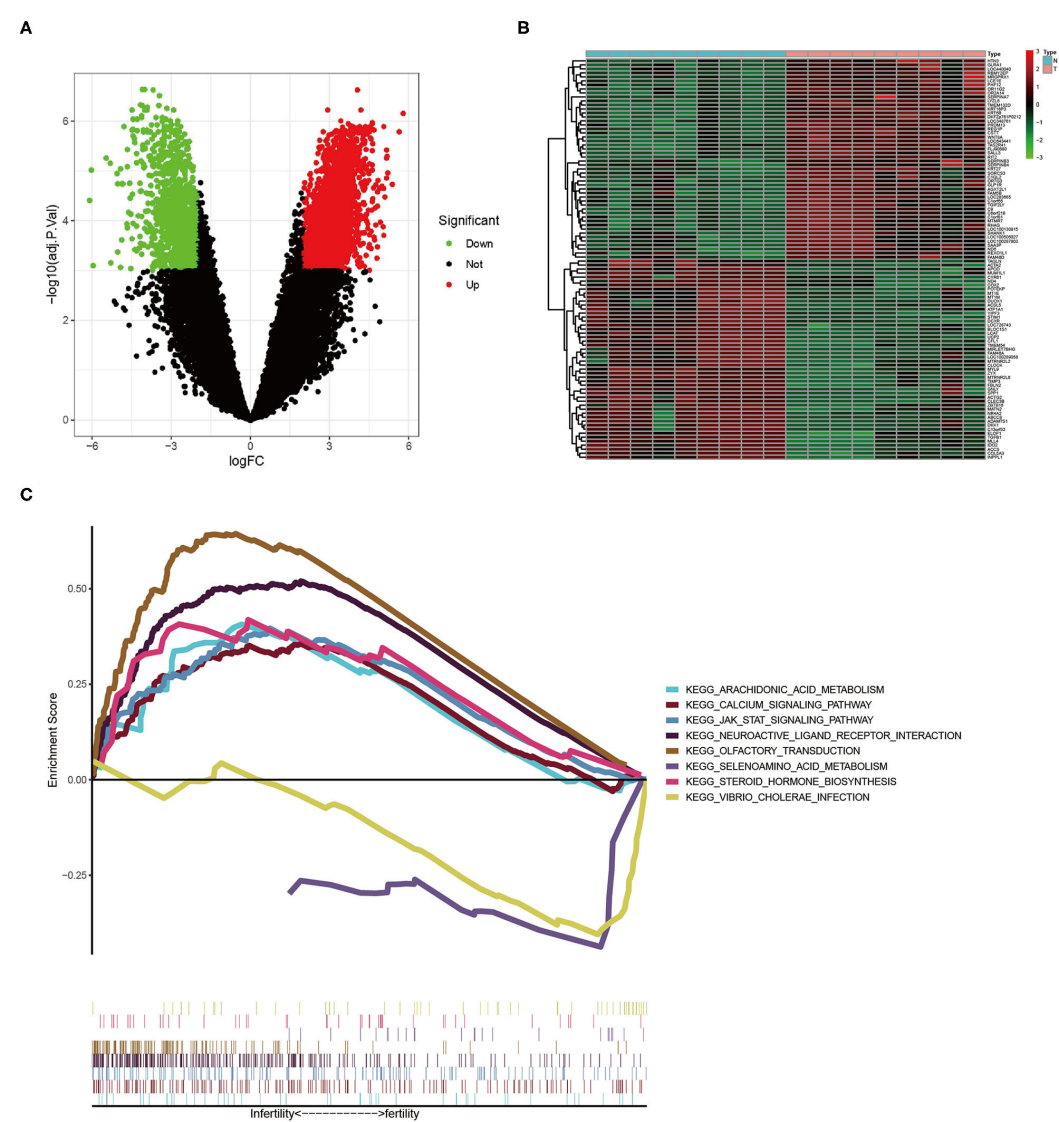
**(A)** The volcano plot of DEGs. **(B)** The heat map of DEGs (only the 50 most up-regulated and down-regulated genes are shown, respectively). **(C)** The results of GSEA enrichment analysis.

Using the “limma” package in R software, we discovered 3,149 differentially expressed genes (Supplementary Table 1, Figure 1A). Low-expressed genes were represented by green dots on the left and high-expressed genes were represented by red dots on the right. We generated the heat map to show the 50 most significantly up-regulated and down-regulated genes in each sample, as shown in Figure 1B. The level of gene expression was linked to color. Low-level expression was represented by green, whereas high-level genes were represented by red. We discovered 8 pathways using GSEA (only showed signaling pathways with *p* < 0.05), including arachidonic acid and selenium amino acid metabolism, calcium signaling pathway, steroid hormone biosynthesis, and JAK-STAT signaling pathway (Figure 1C).

### Results of the WGCNA

**Figure 2:**
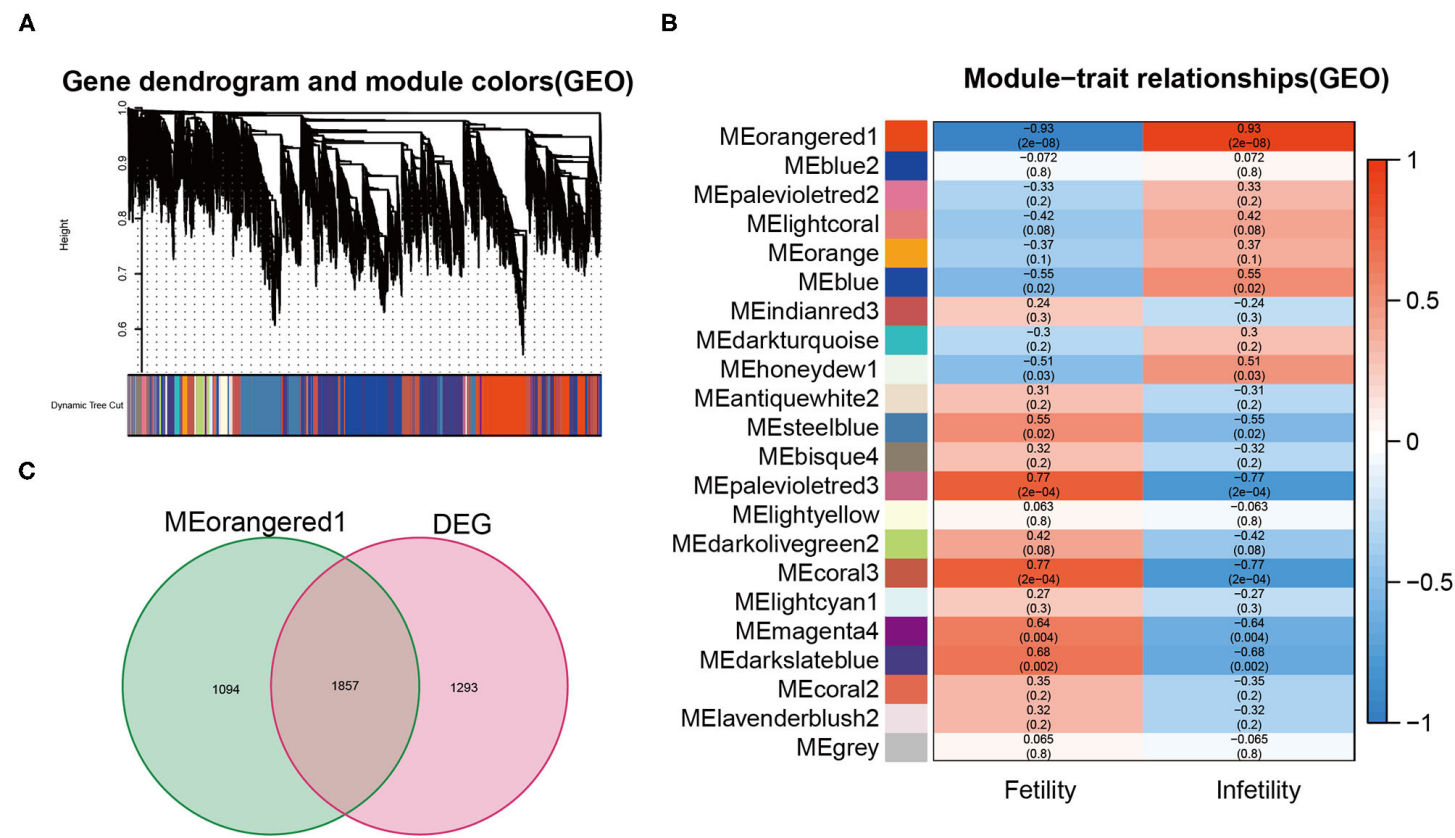
**(A)** The cluster dendrogram of co-expression genes in infertile endometriosis. **(B)** Module–trait relationships in infertile endometriosis. **(C)** The intersection genes between DEG and the orangered1 modules.

WGCNA constructs the weighted co-expression network based on all genes' expression levels obtained by sequencing to indicate the association between gene modules and diseases, as well as the strength of the relationship. 22 co-expressed gene modules were discovered by WGCNA analysis (Figure 2A). As shown in Figure 2B, the MEorangered1 module (Supplementary Table 2) had the strongest relationship with infertile endometriosis (R = 0.93, *p* = 2e-8). As shown in Figure 2C, we integrated the genes in the MEorangered1 module with the DEG to yield 1857 overlapping genes, which we termed gene set 1 (Supplementary Table 3).

### Bioinformatics Analysis of Gene Set 1

**Figure 3:**
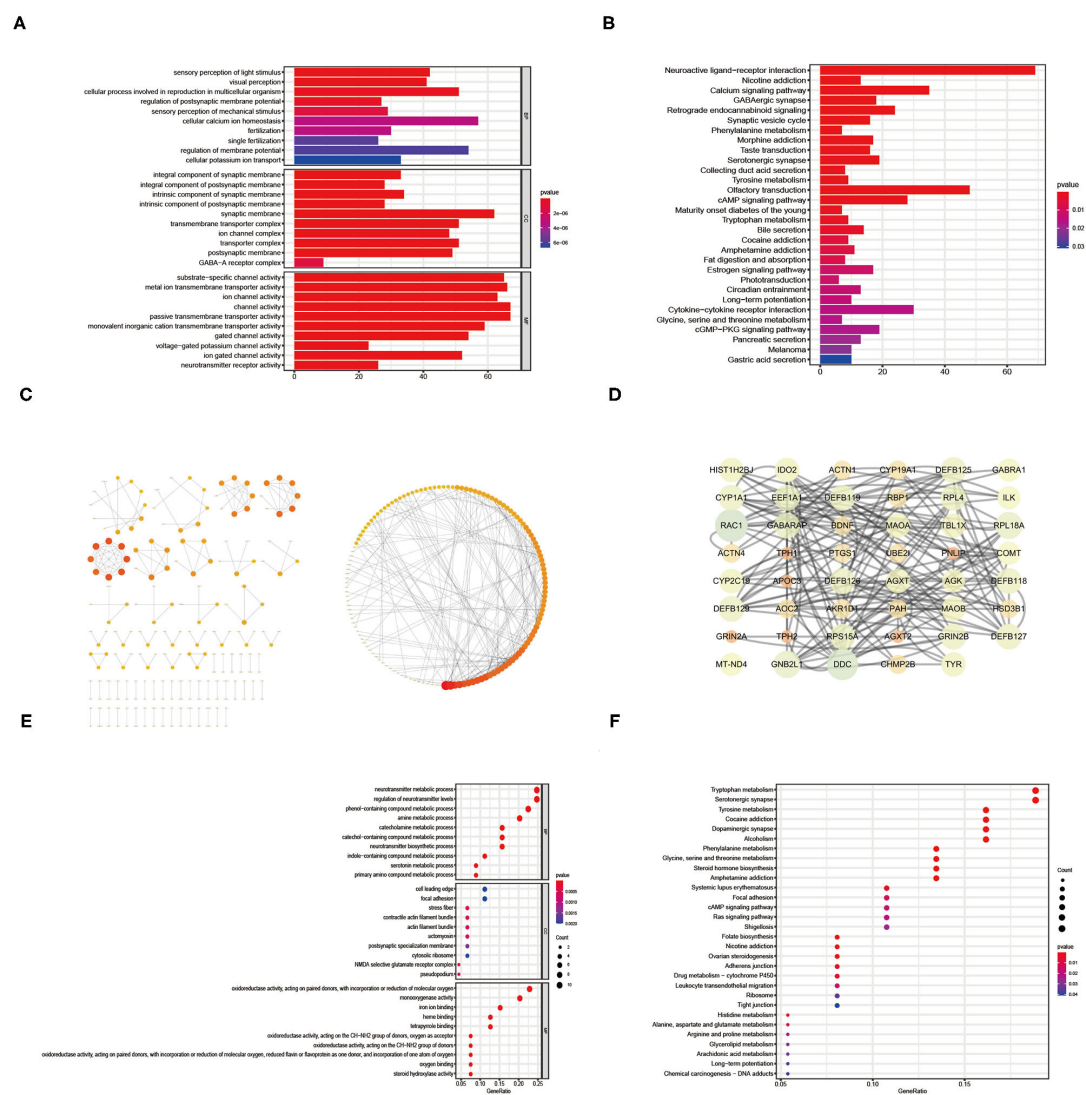
The GO enrichment analysis, KEGG enrichment analysis and PPI network results of intersection genes between DEG and the Meorangered1 modules **(A–C)**. The GO enrichment analysis, KEGG enrichment analysis and PPI network results of hub genes from intersection genes between DEG and the Meorangered1 modules **(D–F)**.

The results of the GO enrichment analysis for gene set 1 were shown in Figure 3A (Supplementary Table 4). In terms of Biological Process, results closely related to infertility include single fertilization, fertilization, and cellular process involved in reproduction in multicellular organisms. In terms of Cellular Component, it was mainly related to postsynaptic, transporter complex, ion channel complex, and transmembrane transporter complex. In terms of Molecular Function, it was mainly related to neurotransmitter receptor activity, ion channel activity, gated channel activity and substrate-specific channel activity. Infertile endometriosis might be linked to amino acid metabolism (Glycine, Serine, Tryptophan, Tyrosine, Phenylalanine, and Threonine), Gastric acid secretion, Bile secretion, Collecting duct acid secretion, cGMP–PKG signaling pathway, cAMP signaling pathway, Calcium signaling pathway, and so on, according to the results of KEGG enrichment analysis (Supplementary Table 5).

### Identification of Hub Genes

Though the STRING database, we generated the PPI network for Gene set 1 with 342 nodes and 397 edges (Figure 3C, Supplementary Table 6). Only non-isolated nodes (linked to at least one node) and edges with gene interaction values > 0.9 are shown in the PPI network. The more genes related with a node, the redder it is. We used cytoscape software to identify nodes with values greater than the median based on mediator, proximity, degree, eigenvector, network, and local average connectivity in order to construct the PPI network (Figure 3D, Supplementary Tables 7, 8). The circle's diameter is proportional to the node's degree.

### Bioinformatics Analysis of Hub Genes

We performed GO enrichment analysis on the hub genes retrieved (Figure 3E, Supplementary Table 9). In terms of biological processes, the findings were mainly related to the metabolic processes of the human body, including serotonin, indole-containing compound, catechol-containing compound, catecholamine, amine, and neurotransmitter etc. In terms of cell components, it was mainly related to cytosolic ribosome, cell leading edge, actomyosin, pseudopodium, etc. In terms of Molecular Function, the results mainly related to oxidoreductase activity, monooxygenase activity, steroid hydroxylase activity, heme binding, oxygen binding. The results of KEGG enrichment analysis found that the cause of infertility in endometriosis may be related to the metabolism of a variety of amino acids (tyrosine, phenylalanine, tryptophan, arginine, proline, etc.), Ras signaling pathway, cAMP signaling pathway, Ovarian steroidogenesis, and so on (Figure 3F, Supplementary Table 10).

### Potential Mechanism of Infertile Endometriosis

**Figure 4:**
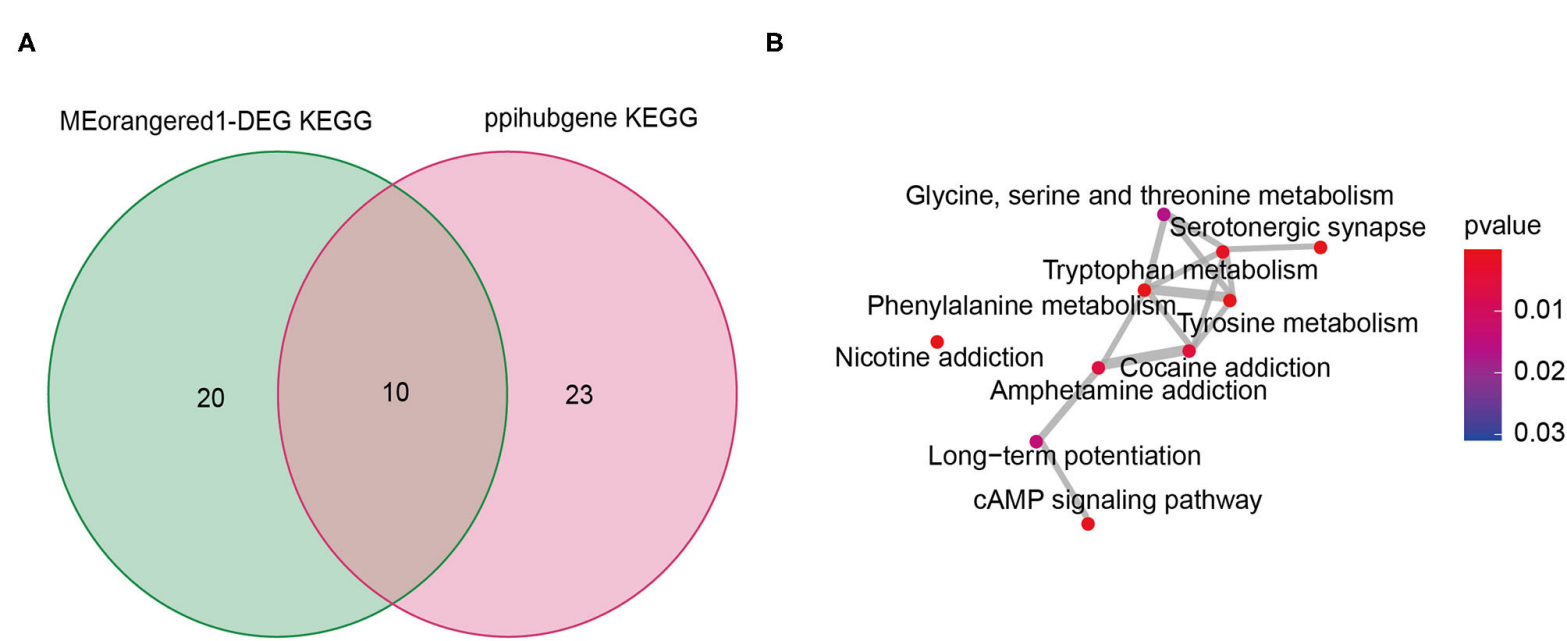
The shared signaling pathway of KEGG enrichment analysis from intersection genes between DEG and the Meorangered1 modules and hub genes **(A)**. Crosstalk analysis of the shared signaling pathway **(B)**.

We intersected the pathways in the KEGG enrichment analysis results of hub genes and gene set 1, and found a total of 10 overlapping pathways (Figure 4A, Supplementary Table 11). The “enrichplot” package in R software was used to identify and illustrate the relationships between these 10 pathways. As shown in Figure 4B, the mechanism of infertile endometriosis might be related to the metabolism of multiple amino acids (Tryptophan, Tyrosine, Phenylalanine, Glycine, serine and threonine), and chemical addiction (Nicotine, Cocaine, Amphetamine), Serotonergic synapse, cAMP signaling pathway, Long-term potentiation.

### Construction of ceRNA Network

**Figure 5:**
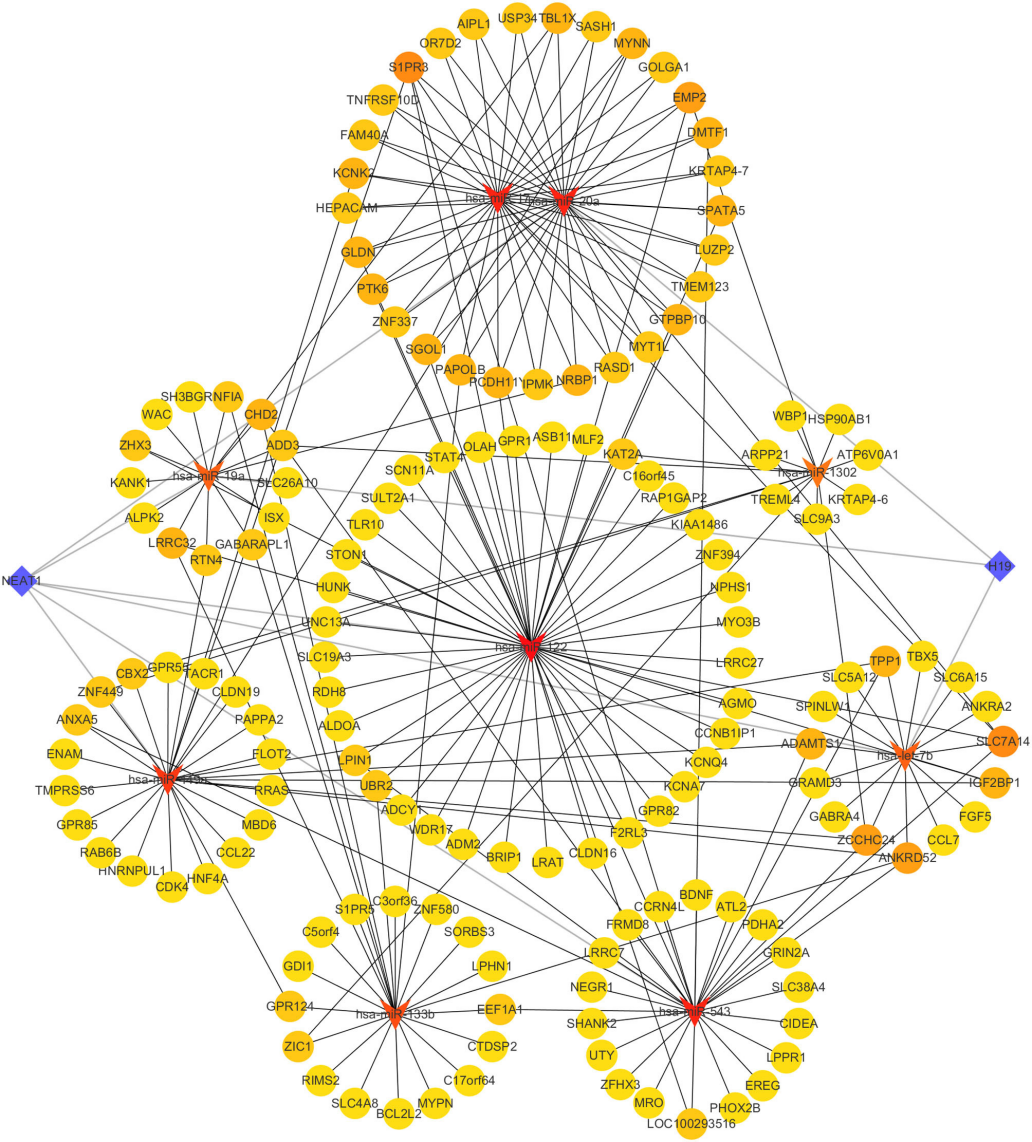
The ceRNA regulatory network. Red triangles, yellow circles, and blue diamonds correspond to miRNAs, hub genes, and lncRNAs, respectively.

Through using HMDD database, we discovered 13 miRNAs related to infertility, including hsa-let-7b, hsa-mir-122, hsa-mir-1302, hsa-mir-133b, hsa-mir-17, and others (Supplementary Table 12). Through the mirTarBase database, the mRNAs controlled by these 13 miRNAs were predicted (Supplementary Table 13). Following that, we crossed each miRNA target gene with gene set 1 (Supplementary Table 14). We discovered two lncRNAs associated to infertility in the LncRNADisease database: H19 and NEAT1. We used the online tool LncBase Experimental v.2 to search for the relationship between infertility-related lncRNAs and miRNAs. Finally, we used Cytoscape software to create the ceRNA regulatory network (Figure 5). mRNA, miRNA, and lncRNA are represented by yellow circle nodes, red triangle nodes, and blue diamond nodes, respectively. The darker the color, the more nodes are connected to the node.

## Discussion

Endometriosis is a non-malignant gynecological disease whose pathophysiology is currently unknown. For the diagnosis of endometriosis, laparoscopy remains the gold standard, and no specific biomarkers or therapeutic targets have been identified (13, 14). It is estimated that about 10% of women of childbearing age suffer from endometriosis. Short menstrual cycles, night work, and short breastfeeding time are high risk factors for endometriosis (13–15). Infertility is one of the most prevalent endometriosis complications, and it has the significant impact on patients' lives. However, the molecular mechanisms by which endometriosis causes infertility are unclear. In this study, we download the patient's transcriptome data and used bioinformatics methods to explore the molecular mechanism and regulatory network between infertility and endometriosis. The results of this study may provide a new research direction for infertility endometriosis and provide help for the clinical treatment of patients.

Through KEGG enrichment analysis of genes set 1, we identified multiple pathways closely associated with infertile endometriosis, including toxic substance addiction (Nicotine, Morphine, Cocaine), estrogen signaling pathways, calcium signaling pathways and metabolism of multiple amino acids (Phenylalanine, Glycine, serine, threonine, Tryptophan, Tyrosine). Smoking during pregnancy has been associated with the development of endometriosis by the mechanism related to smoking-induced changes in maternal estrogen levels (16–18). Nicotine, which is found in cigarette smoke, is one of the chemicals known to be detrimental to human health. Nicotine affects the female reproductive system by suppressing the expression of receptors for steroid hormones in endometrial cells (19). Morphine, a member of the opium alkaloids family, is associated with female reproductive system. The ovaries of rats treated with morphine may develop cystic changes that lead to the development of anovulatory infertility (20). Cocaine may be a risk factor for primary infertility. Women who use cocaine have a considerably higher risk of infertility linked to malformed fallopian tubes than women who do not use cocaine (21). When endometrial tissue forms over other tissues, the body's estrogen signaling pathways are disrupted, leading to pelvic pain and reducing the likelihood of pregnancy (22). One of estrogen's effectors, brain-derived neurotrophic factor, can be utilized as a biomarker to measure female infertility. Brain-derived neurotrophic factor is associated with the proliferation of endometrial tissue and is considered a potential target for infertile patients with endometriosis (23). The calcium signaling pathway is considered to be an important component of human reproduction as it influences the biological processes of cell division, differentiation and death (24, 25). By regulating downstream effectors, the calcium signaling system integrates and decodes information from the cellular microenvironment to mediate biological processes like egg activation and embryonic development (26). There is a strong correlation between enteral nutrition and reproduction. Amino acids in the diet influence key molecules involved in a range of biological processes during conception, such as oocyte fertilization and embryo implantation (27).

PPI network was used to screen the hub genes in gene set 1. To further clarify the molecular mechanisms associated with the development of infertile endometriosis, we intersected the KEGG enrichment results of the hub genes and the genes in gene set 1 to obtain a total of 10 pathways. We believe that these ten pathways are highly relevant to the molecular mechanisms that lead to the onset of infertility in endometriosis. Of these, nicotine addiction and cocaine addiction are recognized risk factors for the emergence of infertility in women (28, 29). Two-fifths of the pathway involves amino acid metabolism and therefore we believe that abnormal amino acid metabolism leads to infertility in patients with endometriosis.

To find potential biomarkers and therapeutic targets for infertile endometriosis patients, the degree values of each node in the PPI network were calculated. The four genes with the highest degree values, DDC, RAC1, GNB2L1, and RPL18A, were identified as possible targets for the identification and therapy of infertile endometriosis. DDC catalyzes the manufacture of dopamine and serotonin, which helps govern neural function. Although DDC is implicated in apoptosis and has been linked to a number of human neurodegenerative illnesses, the mechanisms underpinning its link to infertility are unknown (30, 31). Down-regulation of RAC1 expression in mouse endometrium leads to embryo implantation failure (32). Through the AKT/NF-kappaB pathway, RAC1 is involved in inflammatory changes in endometrial tissue, which is a common cause of female infertility (33).

Endometriosis is a benign disease, but it has the ability to invade and maintain the survival function of ectopic tissue, similar to cancer. RPL18A is involved in the cycle arrest process of non-small cell lung cancer A549 cells by affecting the expression of several cell cycle-related proteins, including Cyclin A2 and Cyclin B1 (34). The expression of GNB2L1 was higher in ectopic endometrial tissue from patients with endometriosis than in normal patients, regardless of whether they were in the proliferative or secretory phase of the menstrual cycle. GNB2L1 is associated with the progression of endometriosis and contributes to the removal of ectopic endometrium by reducing cell proliferation and angiogenesis (35). Therefore, GNB2L1 has the potential to act as a biomarker and therapeutic target for endometriosis. These four genes may provide assistance in the clinical detection and treatment of infertility in patients with endometriosis.

Non-coding RNAs (ncRNAs) are not directly involved in protein translation, but they are involved in many biological processes in the body (36). ncRNAs are associated with the development of many diseases and can be divided into small RNAs and lncRNAs depending on their length. LncRNAs have functions as molecular scaffolds for chromatin and miRNA sponges, translating and degrading RNAs that are involved in the development of most diseases (37). As a type of small ncRNA, miRNA regulates gene expression mainly by silencing mRNA translation and degrading mRNA. LncRNA control gene expression by binding to miRNAs in a competitive manner. As a result, the ceRNA network connects the mRNAs, miRNAs, and lncRNAs, contributing to our understanding of the gene regulatory network and molecular mechanisms that lead to infertility in endometriosis patients. We searched the HMDD, mirTarBase database, and LncRNADisease database for miRNAs and lncRNAs linked to infertility, and then used Cytoscape software to build a ceRNA regulatory network linked to infertile endometriosis. This ceRNA network can reveal the regulatory links between genes linked to infertility, as well as biomarkers and targets for the diagnosis and therapy of infertile endometriosis.

## Conclusion

In this study, we included sequencing results of endometrial tissue from fertile and infertile patients with endometriosis. We discovered that the mechanism of infertile endometriosis patients may be linked to amino acid metabolism by bioinformatics analysis. We also constructed the ceRNA regulatory network that could be linked to infertility in women with endometriosis. These findings could aid in the clinical treatment of patients.

## Data Availability Statement

The original contributions presented in the study are included in the article/Supplementary Materials, further inquiries can be directed to the corresponding authors.

## Author Contributions

WL, HW, XL, and CY developed the concept of the project and wrote the manuscript. XL and LG collected and analyzed the data with the help of WZ, JH, and LA. All authors reviewed and discussed the results and contributed to the paper preparation. All authors have read and approved the manuscript.

## Funding

This study was supported by Shenzhen Key Laboratory Foundation (ZDSYS20200811143757022) and Shenzhen Science and Technology Innovation Commission Project (Grant No. JCYJ20180302174235893).

## Conflict of Interest

The authors declare that the research was conducted in the absence of any commercial or financial relationships that could be construed as a potential conflict of interest. The handling editor YF declared a shared affiliation with the author CY at the time of the review.

## Publisher's Note

All claims expressed in this article are solely those of the authors and do not necessarily represent those of their affiliated organizations, or those of the publisher, the editors and the reviewers. Any product that may be evaluated in this article, or claim that may be made by its manufacturer, is not guaranteed or endorsed by the publisher.

## Supplementary Material

Supplementary Table 1 DEGs.

Supplementary Table 2 The genes in MEorangered1 module.

Supplementary Table 3 The genes in gene set 1.

Supplementary Table 4 The GO enrichment analysis results of gene set 1.

Supplementary Table 5 The KEGG enrichment analysis results of gene set 1.

Supplementary Table 6 The PPI network of gene set 1.

Supplementary Table 7 Calculation results of betweenness, closeness, degree, eigenvector, network and local average connectivity-based method.

Supplementary Table 8 The hub genes.

Supplementary Table 9 The GO enrichment analysis results of hub genes.

Supplementary Table 10 The KEGG enrichment analysis results of hub genes.

Supplementary Table 11 Intersection of KEGG enrichment analysis results of hub genes and gene set 1.

Supplementary Table 12 MiRNA related to infertility.

Supplementary Table 13 Intersection of predicted miRNA target gene and gene set 1.

Supplementary Table 14 MiRNA-mRNA network.

## Abbreviations

GEO: Gene Expression Omnibus
WGCNA: Weighted Gene Co-Expression Network Analysis
DEGs: differentially expressed genes
GO: Gene Ontology
KEGG: Kyoto Encyclopedia of Genes and Genomes
PPI: protein protein interaction
GSEA: Gene Set Enrichment Analysis
miRNA: microRNA
ceRNA: competing endogenous RNAs
HMDD: Human MicroRNA Disease Database
TOM: topological overlap matrix
ncRNAs: Non-coding RNAs.
